# Individualized breathing trace quality assurance for lung radiotherapy patients undergoing 4DCT simulation

**DOI:** 10.1002/acm2.13929

**Published:** 2023-02-20

**Authors:** James Rijken, Yunfei Hu, Kelvin Hiscoke

**Affiliations:** ^1^ Icon Cancer Centre Windsor Gardens South Australia Australia; ^2^ Icon Cancer Centre Gosford New South Wales Australia; ^3^ Icon Cancer Centre Wellington New Zealand

**Keywords:** 4DCT, breathing trace, lung, RGSC, RPM, SABR, VXP

## Abstract

4DCT simulation is a popular solution for radiotherapy simulation of lung cancer patients as it allows the clinician to gain an appreciation for target motion during the patient breathing cycle. Resultant binning of images and production of the 4DCT dataset relies heavily on the recorded breathing trace; but quality assurance is not routinely performed on these and there lacks any substantial recommendations thereof.

An application was created for Windows in C# that was able to analyze the VXP breathing trace files from Varian RPM/RGSC and quantify various metrics associated with the patient breathing cycle. This data was then used to consider errors in voluming of targets for several example cases in order to justify recommendations on quality assurance.

For 281 real patient breathing traces from 4DCT simulation of lung targets, notable differences were found between RGSC and application calculations of phase data. For any new patient without individualized QA, the average marked phase calculation (which is used for 4DCT reconstruction) is only accurate to within 19% of the actual phases. The error in BPM within the scan due to breathing rate variation is 37%. The uncertainty in amplitude due to breathing variation is 34% in the mean. Phase uncertainty leads to misbinning which we have shown can lead to missing 66% of the target for gated treatment. Variation in inhalation/exhalation level leads to voluming errors which, without individualized QA, can be assumed to be 11% (PTV is smaller than actual).

Without individualized quality assurance of patient breathing traces, large uncertainties have to be assumed for metrics of both phase and amplitude, leading to clinically significant uncertainties in treatment. It is recommended to perform individualized quality assurance as this provides the clinician with an accurate quantification of uncertainty for their patient.

## 1 INTRODUCTION

Accurate tumor definition is an important factor in radiotherapy, especially stereotactic body radiotherapy (SBRT) where high ablative doses and small margins are utilized. Tumors located in the thorax and abdomen and sometimes even the pelvis are greatly affected by respiratory motion which needs to be accounted for during treatment delivery.^1^ Incorrect determination of the tumor and its associated motion could result in underdosage of the tumor and unnecessary irradiation of surrounding normal tissue.

An established solution for acquiring high‐quality CT data in the presence of respiratory motion is four‐dimensional computer tomography (4DCT). 4DCT involves the acquisition of a series of 3D CT images over multiple respiratory cycles which are retrospectively sorted, using a surrogate signal, into selected phases of the respiratory cycle either through phase binning or amplitude binning.^2^ Although several motion management techniques in radiation oncology are available, 4DCT remains an essential component of multiple motion‐encompassing and prospective/retrospective gating methods to determine either the entire tumor motion or a portion of it. For example, motion‐encompassing techniques that utilize 4DCT ensures the entire tumor‐motion‐encompassing volume is captured either through contouring the clinical target volume (CTV) on each bin or delineating the composite target volume displayed in the maximum intensity projection (MIP) reconstruction. Alternatively, one of the gated techniques, the mid‐ventilation approach, uses the information on tumor movement from phase‐based 4DCT scans to calculate the time‐weighted mean position of the tumor, or the so‐called mid‐position, followed by choosing the breathing phase from the 4DCT closest to the mid‐position as the basis for the treatment plan.^3^, ^4^, ^5^

A limitation of 4DCT is that it is affected by variations in respiratory patterns during acquisition. Intra‐phase residual tumor motion experienced in cine (step‐and‐shoot) 4DCT can produce partial projection artifacts and is found to be greatest near mid‐ventilation where the tumor velocity is at its maximum.^6^ Artifacts because of breathing irregularities are principally due to incorrect sorting of the individual 3D CT images and have been shown to affect the size and shape of the target volume.^7^, ^8^, ^9^ While patient training has been demonstrated effective in improving breathing reproducibility and reducing breathing irregularity, patients’ breathing patterns can still vary in magnitude, period, and regularity during imaging and treatment sessions. Therefore, it is of great significance to accurately determine the patient's breathing trace on a daily basis.

For both amplitude‐ and phase‐binning 4DCT, the breathing trace generated during image acquisition is critical for the subsequent accurate delineation of the tumor‐motion‐encompassing volume. A common approach to acquiring this breathing trace is through the monitoring of an external surrogate, one example of which is the Varian Respiratory Gating for Scanners (RGSC) system (Varian Medical Systems, Palo Alto CA, USA). In clinical practice, although routine quality assurance (QA) may be performed on the RGSC system itself, individual QA on patients’ breathing traces used to reconstruct 4DCT images during both imaging and treatment is significantly underdeveloped. Some studies have attempted to identify the magnitude of error in the breathing traces generated by RGSC and reported concerning results. For example, a study by Shi et al. indicated that the RGSC system only showed a 76% agreement with the programmed test data within ± 5% tolerance in terms of fitting period.^10^ Another study suggested that irregular breathing could almost double the dose difference to planned dose when compared with delivery from a regular breathing trace.^11^ A phantom study that compared the MIP‐based ITVs from 4DCT to those from dMRI showed that the errors in the former correlated linearly with the subjects’ respiratory variability, and therefore using a 4DCT MIP image to define the ITV might cause underdosing.^12^ However, most of the existing studies are based on phantom measurements and therefore can quantify neither inter‐fractional motion variability of the same patient nor breathing‐trace differences among different patients. In addition, none of the studies have provided individual QA solutions for breathing traces generated by commercial systems such as RGSC, nor measures that can be taken if errors in the breathing trace have been identified.

In this study, an application was created for in C# that was able to analyze the VXP breathing trace files from Varian RPM/RGSC systems and quantify various metrics associated with the patient breathing cycle. The application was subsequently adopted to analyze the breathing trace of 281 clinical patients before treatment planning. The data were then used determine dosimetric error for several examples to justify recommendations on quality assurance.

## 2 METHOD

In order to collect and analyze breathing trace data from 4D‐CT simulations by RGSC/RPM (hereafter just referred to as RGSC), a separate application was written in C#. Radiotherapists took simulation scans with RGSC, reviewed and edited the peak/troughs as required using the RGSC software and uploaded the RGSC produced *.VXP (or *.DAT for much older systems) file to the application at the time of simulation and the breathing trace was analyzed, with results saved to a central *.CSV file for later analysis. Data was collected over the course of a year from CT scanners associated with 25 radiotherapy sites across Australia and New Zealand. In total, breathing traces from 281 lung patient 4D‐CT simulations were acquired. This was a retrospective study on completely anonymous data.

**FIGURE 1:**
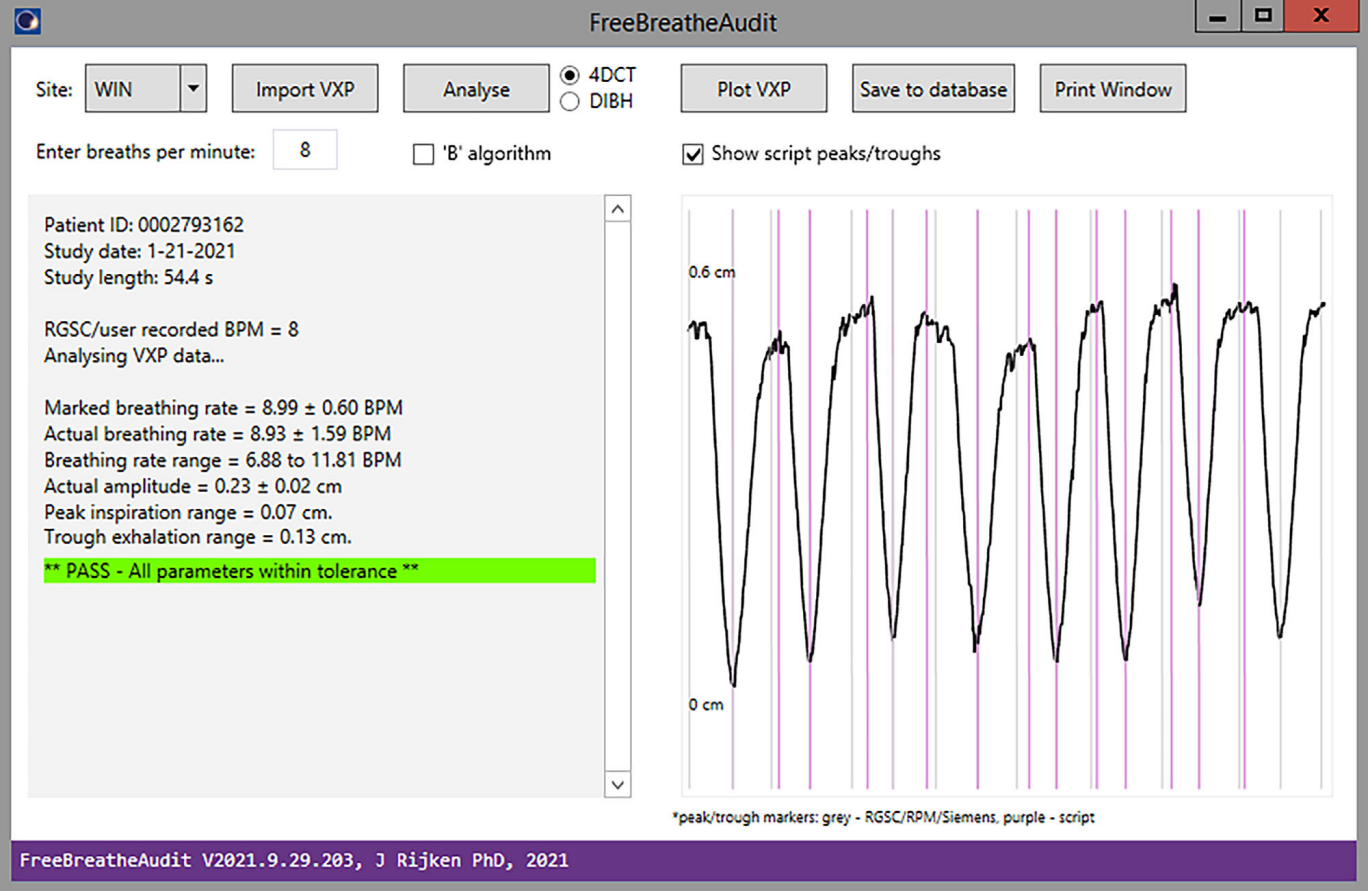
FreeBreatheAudit application window for an example patient showing data collected and calculated from the patient's breathing trace as well as the amplitude plot with peaks and troughs marked in purple (calculated in application) and black (taken from *.VXP file).

The C# application, called FreeBreatheAudit, consisted of a simple graphical user interface that allowed the user to input the nominal breaths per minute as shown in the RGSC window. Results were printed in the application window and a visual representation of the breathing trace with marked peaks and troughs was also displayed. This is shown in Figure 1 for an example patient scan.

As shown in Figure 1, several metrics were calculated for each breathing trace. These are described as follows:

*Marked breathing rate* is the average value of breaths per minute (BPM) calculated from the time between each ‘P’ or ‘Z’ marking in the *.VXP file indicating where RGSC has placed a peak or trough. Peak and trough placement is automatic (sometimes manually edited in RGSC or Siemens workstation). The marked peaks/troughs may be different to the actual (‘actual’ being the ones calculated in our application algorithm) peaks/troughs. This metric was reported for comparison with subsequent metrics. The error reported was the standard deviation in the sample of BPM values calculated for each peak‐trough interval.

*Actual breathing rate* is the average BPM value calculated from the time between each actual local peak and trough (hereafter referred to as a ‘phase’) in the breathing trace. Peaks and troughs were algorithmically located and ignored small fluctuations and noise that did not contribute to the breathing cycle. Data were smoothed before analysis with a moving average. This metric was reported to check for gross errors in marked values and marked breathing rate which is used for 4DCT binning. The error reported was the standard deviation in the sample of BPM values calculated for each phase interval.

*Breathing rate range* gives the lowest and highest calculations of BPM from each phase along the breathing trace. This shows whether the patient breathing is too variable, which is also demonstrated by the standard deviation in marked/actual breathing rates.

*Actual amplitude* is the average of the amplitudes calculated at each phase. The error reported is the standard deviation in the sample of amplitudes for a given scan.

*Peak inspiration and trough exhalation range* are the largest differences between the lowest and tallest respective peaks and troughs. This gives an appreciation as to whether the patient is taking progressively deeper or shallower breaths as the simulation scan progresses or whether there is no consistency in spirometry.

In addition to these, the “GUI” BPM was also recorded, which is the breathing frequency shown on the RGSC user interface. This is the value typically entered into some CT software to assist it in 4DCT reconstruction and binning.

These metrics were collected and analyzed for the whole patient sample. The calculated errors expected in a patient breathing trace were used then to infer whether individualized quality assurance of patient breathing traces was warranted. To illustrate the impact of any expected uncertainty calculated from the analysis of patient breathing traces, underdosing was calculated for hypothetical but realistic patient plan scenarios. This was based upon the calculated uncertainties in both the breathing phase and amplitude.

Firstly, the phase uncertainty was calculated. For gated (or phase based) treatment, the effect of phase‐based error can be clinically significant since the target will be binned on an incorrect phase (other techniques like MIP/average 4DCTs and breath hold are insensitive to this). Positional uncertainties can be estimated based on a possible phase shift calculated from the phase uncertainty, assuming a simple cosine curve breathing trace (phase 0%−10% is the 1st peak in the cycle). The difference in target position is then given by

$$\Delta y=A\left(\cos\left(x\times 2\pi \right)-\cos\left(\left(x+\varphi \right)\times 2\pi \right)\right)$$

where *A* is the amplitude (half the distance between peak and trough), *φ* is the resultant phase shift and *x* is the breathing phase as a percentage. Differences in tumor position for prospective gating due to uncertainties in BPM variation were calculated for a range of breathing amplitudes. This was then applied as a worst‐case scenario to a realistic clinical case of SBRT lung treatment with a PTV of 2 cm diameter, tumor motion of 2 cm,^1^, ^13^, ^14^ and treated at mid‐ventilation.^3^ ^‐^ ^5^

In the next clinical application, the impact of amplitude variability on voluming of the target was calculated according to the methods of Cai et al.^12^ Voluming error *ϵ* is taken from the amplitude variability: *ϵ* = −5.13 v− 6.71, *r* ^2^ = 0.76 where *v* = (SD(peaks)+SD(troughs))/2 [mm].^12^ It can also be expressed in terms of internal target area (ITA) differences:

$$\varepsilon =\frac{\mathrm{IT}A_{\mathrm{sim}}-\mathrm{IT}A_{\mathrm{real}}}{\mathrm{IT}A_{\mathrm{sim}}}$$

so we can estimate the added margin required, *m*, due to voluming error to the MIP from simulation:

$$m=\frac{-\varepsilon \times x_{\mathrm{MIP}}}{2}$$

where *x*_MIP_ is the length of the MIP in the direction of motion. Amplitude variability was taken from the resultant mean from the sample of per scan standard deviations in amplitude. Patient cases were considered with varying MIP lengths. The amount of real ITA compared with that drawn up from simulation informs the extent of target volume not being exposed.

Finally, patient case studies were identified that had slipped through the process of performing individualized breathing trace QA before planning. These cases help to demonstrate the need for individualized QA of breathing traces.

## 3 RESULTS

### 3.1 Data acquisition and analysis

**FIGURE 2:**
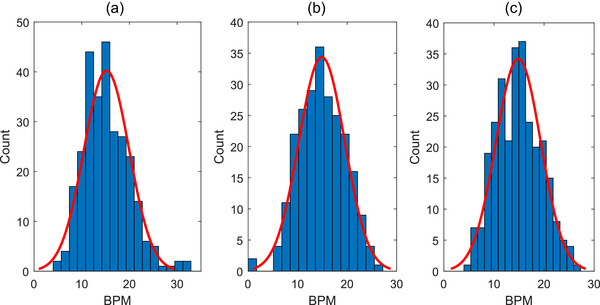
Average BPM values for the patient sample taken from (a) RGSC GUI, (b) Marked BPM, and (c) Actual BPM (*n* = 281).

For the sample of 281 lung patients from 25 centers across Australia and New Zealand, average BPM is shown in Figure 2. Gaussian distribution curves were fitted using MATLAB's statistical package (MATLAB R2019a, MathWorks, Natick MA, USA) with mean and standard deviation values shown in Table 1. Both marked and actual BPM distributions did not show the higher values represented in the RGSC GUI BPMs. As a result, the mean BPM values for both marked and actual samples are slightly lower than for the GUI sample and the standard deviations are also smaller. As a whole, all three methods had good agreement with each other for calculating the patient average BPMs. For lung patients with no breath coaching, the mean breathing rate on CT simulation was approximately 15 BPM with a standard deviation of 4.6 BPM. This means that 95% of patients had a breathing rate between 6 and 24 BPM, demonstrating the large variability in patient breathing.

**TABLE 1:** Gaussian distribution parameters for spread of BPM data shown in Figure 2 with 95% confidence intervals.

Method	*μ*	*σ*
GUI BPM	15.2 (14.6, 15.8)	4.7 (4.4, 5.2)
Marked BPM	14.9 (14.3, 15.5)	4.6 (4.2, 5.1)
Actual BPM	14.9 (14.3, 15.4)	4.5 (4.1, 4.9)

**FIGURE 3:**
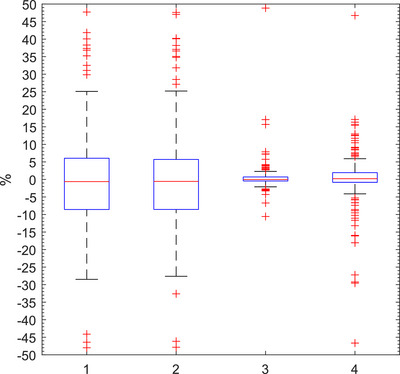
Complete sample of per scan differences between (1) marked BPM and GUI BPM, (2) actual BPM and GUI BPM, (3) actual BPM and marked BPM, and (4) standard deviation in actual BPM and marked BPM (*n* = 281).

The difference between GUI BPM, Marked BPM and Actual BPM was calculated for each scan. This is shown for the sample of patients in Figure 3 in the form of a box and whisker plot. The difference between marked BPM and the GUI BPM was on average only −0.5% but had a standard deviation of 20.1%. The difference between the actual BPM and the GUI BPM also had a mean close to zero (−0.4%) and a standard deviation of 18.6%. The difference between marked and actual BPM was 1.3% ± 9.6% (1 SD) demonstrating the uncertainty in RGSC's placement of peaks and troughs on the breathing cycle. The standard deviation in BPM was calculated using the ‘marked’ and ‘actual’ methods and this was compared for each scan (Figure 3(4)). The average difference in breathing variation as calculated by the two methods was 2.9% ± 18.4% (1 SD) with marked BPM showing a greater variance. These differences are summarized in Table 2.

**TABLE 2:** Average differences between parameters for a given scan as determined by “GUI,” “marked,” and “actual” methods (*n* = 281).

Parameter	Method A	Method B	Difference (1 SD)
BPM mean	GUI	Marked	−0.5% ± 20.1%
BPM mean	GUI	Actual	−0.4% ± 18.6%
BPM mean	Marked	Actual	1.3% ± 9.6%
BPM SD	Marked	Actual	2.9% ± 18.4%

**FIGURE 4:**
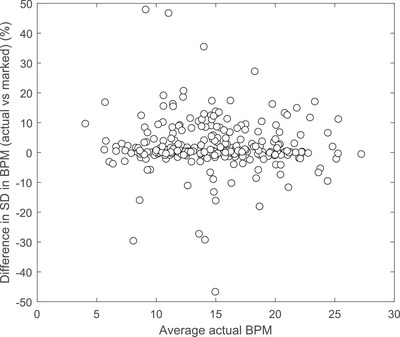
Difference in standard deviation in BPM (marked vs. actual) plotted against actual average BPM (outliers not shown) (*n* = 281).

The differences in BPM variation were plotted against average BPM and were found to be completely uncorrelated (Figure 4)

**FIGURE 5:**
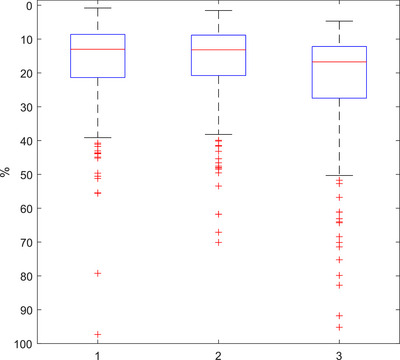
Per scan standard deviation in (1) marked BPM, (2) actual BPM, and (3) amplitude (*n* = 281).

The standard deviation in BPM for each patient scan is shown in Figure 5 for both ‘marked’ and ‘actual’ calculation methods. The overall distribution of standard deviations for both these methods appears in close agreement but Figure 3(4) demonstrates that there are notable differences when comparing each method for individual scans. The average standard deviation observed was 18% for both methods when including outliers.

**FIGURE 6:**
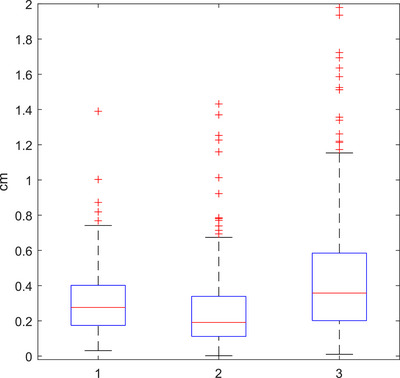
Per scan (1) average amplitude, (2) peak range, and (3) trough range (*n* = 281).

Amplitude data were also collected for each scan based on the actual peaks and troughs. The sample of amplitude calculations per scan was used to calculate the standard deviation in amplitude per scan. This is shown in Figure 5(3) where patient scans were most likely to vary in amplitude by 17%. The distribution of average amplitudes for all scans is shown in Figure 6(1). Most patients have a chest/abdomen amplitude of around 0.3 cm (0.6 cm from peak to trough). Most surprisingly, the amount of variation in the peak or trough position per scan was equal or greater to the average amplitude, thus demonstrating the uncertainty in patient spirometry. Most patients had a peak and/or trough variation of around 0.2–0.4 cm over the course of their scan. This is shown in Figure 6(2) and 6(3), with many patients having variation in inhalation or exhalation of up to 1.2 cm.

**FIGURE 7:**
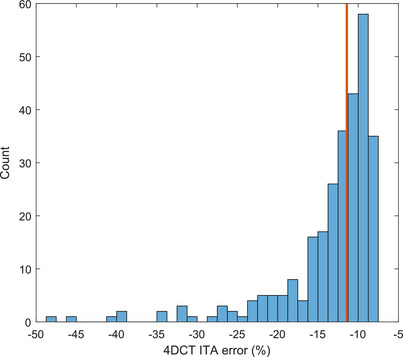
ITA voluming error per patient based on variability in breathing amplitude.^12^ The median value of 11.41% is shown in red. (*n* = 281).

The distribution of ITA errors, based on the calculation methods of Cai et al.^12^ is shown in Figure 7. The distribution of error is non‐gaussian with a median value of −11.4%. Non‐outlier error for the patient sample was between −7.5% and −22.5%.

From the calculation of these results, based on a large cohort of real patient data, uncertainties to a confidence level of 95% were well defined for any new lung patient being simulated with the use of RGSC for 4DCT reconstruction. For our new patient, BPM displayed on the GUI for the patient breathing is within 39% of the actual average BPM. The average marked BPM (or phase calculation) which is used for 4DCT reconstruction is within 19% of the actual BPM (or phases). BPM variation is on average actually better than the marks indicate, by almost 3%, but the error in BPM within the scan due to breathing rate variation is 37%. The uncertainty in amplitude due to breathing variation is 34% in the mean, with uncertainty in the peak and trough levels of 7 and 12 mm, respectively. The uncertainties in amplitude imply that the ITA will most likely be undervolumed by around 11% leading to underdosing of the target.

### 3.2 Hypothetical cases and patient impact

Data acquired in this study demonstrates that for a given patient scan, the variation in BPM is different between the RGSC marks used for binning and that calculated in this work. Based on our patient study, the uncertainty in this difference is ± 19% (2 SD). Relative uncertainties in breathing rate per breathing cycle are also the uncertainties in the breathing period. This translates to differences in phase where images are binned. So adopting the uncertainty as a hypothetical worst case scenario, a period that is 19% shorter or longer will lead to misbinning by a figure of 2, meaning that images meant for the 0%−10% bin will end up in the 20%−30% bin. Misbinning is unrelated to the average BPM since this has been shown to be uncorrelated (Figure 4). Results of target position differences for a sample of possible tumor amplitudes are given in Table 3.

**TABLE 3:** Difference in tumor position (cm) for prospective gating due to a uncertainties in BPM variation of 19%.

	Phase									
Amplitude (cm)	0%	10%	20%	30%	40%	50%	60%	70%	80%	90%
0.25	0.1	0.3	0.3	0.2	0.0	−0.1	−0.3	−0.3	−0.2	0.0
0.50	0.3	0.5	0.5	0.3	0.0	−0.3	−0.5	−0.5	−0.3	0.0
0.75	0.4	0.8	0.8	0.5	0.0	−0.4	−0.8	−0.8	−0.5	0.0
1.00	0.6	1.0	1.1	0.7	0.1	−0.6	−1.0	−1.1	−0.7	−0.1
1.25	0.7	1.3	1.3	0.9	0.1	−0.7	−1.3	−1.3	−0.9	−0.1
1.50	0.9	1.5	1.6	1.0	0.1	−0.9	−1.5	−1.6	−1.0	−0.1
1.75	1.0	1.8	1.8	1.2	0.1	−1.0	−1.8	−1.8	−1.2	−0.1
2.00	1.2	2.0	2.1	1.4	0.1	−1.2	−2.0	−2.1	−1.4	−0.1
2.25	1.3	2.3	2.4	1.5	0.1	−1.3	−2.3	−2.4	−1.5	−0.1
2.50	1.5	2.6	2.6	1.7	0.1	−1.5	−2.6	−2.6	−1.7	−0.1
2.75	1.6	2.8	2.9	1.9	0.2	−1.6	−2.8	−2.9	−1.9	−0.2
3.00	1.8	3.1	3.2	2.1	0.2	−1.8	−3.1	−3.2	−2.1	−0.2

The extent of mistreatment of the target and normal tissue due to phase‐based error depends on multiple factors, including the tumor size, tumor movement amplitude, planning margin, breathing pattern, plan quality, etc. Due to the complexity, there is currently no literature that has quantified this effect. However, in this study, an example estimation is provided based on the following case assumptions:

- Breathing trace = standard cosine curve
- PTV size = 2 cm diameter circle
- Tumor motion = 2 cm^1^, ^13^, ^14^
- PTV—CTV/GTV margin = 0.5 cm
- Mid ventilation using the 20%–40% breathing phase for treatment^15^ (assumption as in Table 3 where 0% is fully inhaled)

**FIGURE 8:**
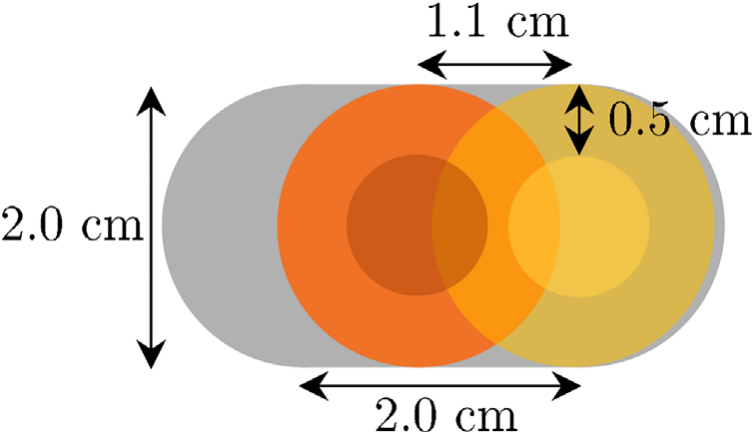
Graphical representation of the difference in tumor position (red is intended position and yellow is actual position) for our example case where a 2 cm diameter target with a 2 cm tumor motion has a phase offset of 19%. This equates to a positional variation of 1.1 cm when the intended phase was mid‐ventilation (20%–40%). GTV/CTVs are shown as the smaller non‐overlapping circles.

In this case, with a phase binning error of 19% applied consistently throughout the scan as a worst‐case scenario supported by our results, the treatment actually happens at the 40%–60% phases rather than the initial 20%–40% phases. According to Table 3, the difference in tumor position with a 1 cm amplitude will be up to 1.1 cm as shown in Figure 8. The overlap area is the PTV area treated which is 1.06 cm^2^ (2 × segment area). As a result, even with the 0.5 cm margin, the amount of PTV missed during treatment is (3.14−1.06)/3.14 = 66%. This possibly could occur during treatment at our center since the same software (RGSC) is used for gating during treatment as with simulation.

Lastly, voluming error based on amplitude variation^12^ for example MIP lengths was calculated. The required ITV extensions in the direction of motion at both the inhale and exhale positions are shown in Table 4, based upon the median voluming error, *ϵ* = −11.4% calculated from our patient study.

**TABLE 4:** Additional voluming required in the direction of motion at both the inhale and exhale positions based on the median voluming error of −11.4% demonstrating in our patient study and also the methods of Cai et al .^12^

MIP length (cm)	Missed tissue at both ends of travel (mm)
2.0	1.1
3.0	1.7
4.0	2.3
5.0	2.9
6.0	3.4
7.0	4.0

### 3.3 Case studies

#### 3.3.1 Case 1

**FIGURE 9:**
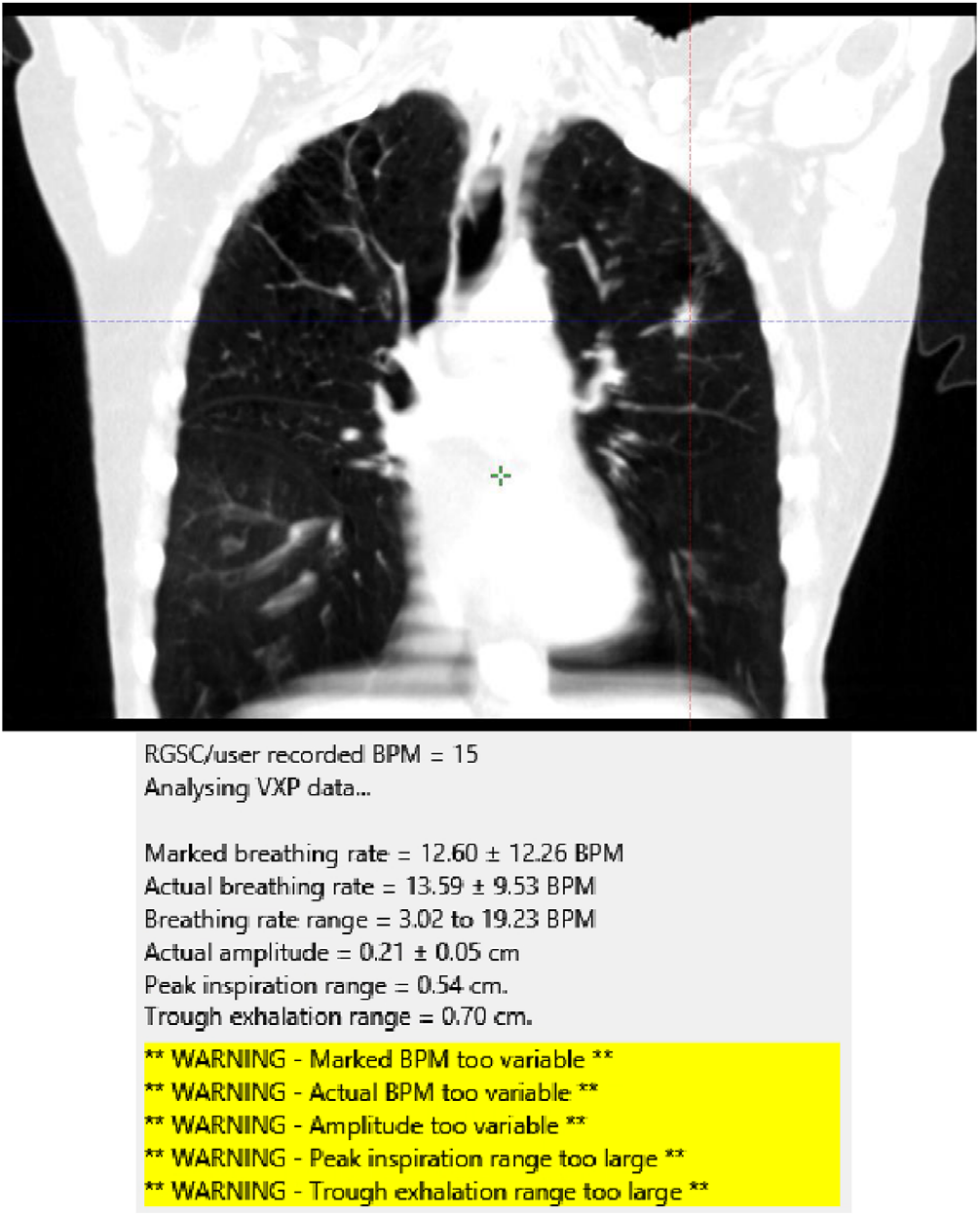
Case 1 example of poor 4DCT reconstruction with retrospective fail with FreeBreatheAudit. Breathing trace exceeded statistical tolerance (1 SD) for a number of metrics.

The first patient case that was identified at planning as having a poor 4DCT reconstruction had slipped through our process and had not had FreeBreatheAudit run after simulation while the patient was still on the CT couch. FreeBreatheAudit was run retrospectively to see if this case would have been identified for re‐simulation. A coronal view of the dataset and the failed report are shown in Figure 9. The reconstructed dataset had artifacts for the full length of scan with many areas of inconsistent HU. This patient had a difference of average actual to marked frequency of 7% and a difference in standard deviation of 27%, the difference between marked and actual phases resulting in images binned in the wrong phase. Error in amplitude was 24%. This case would have immediately flagged as a fail (tolerances based on statistical uncertainty 1 SD), requiring a re‐simulation. This highlights the need for such QA measures.

#### 3.3.2 Case 2

**FIGURE 10:**
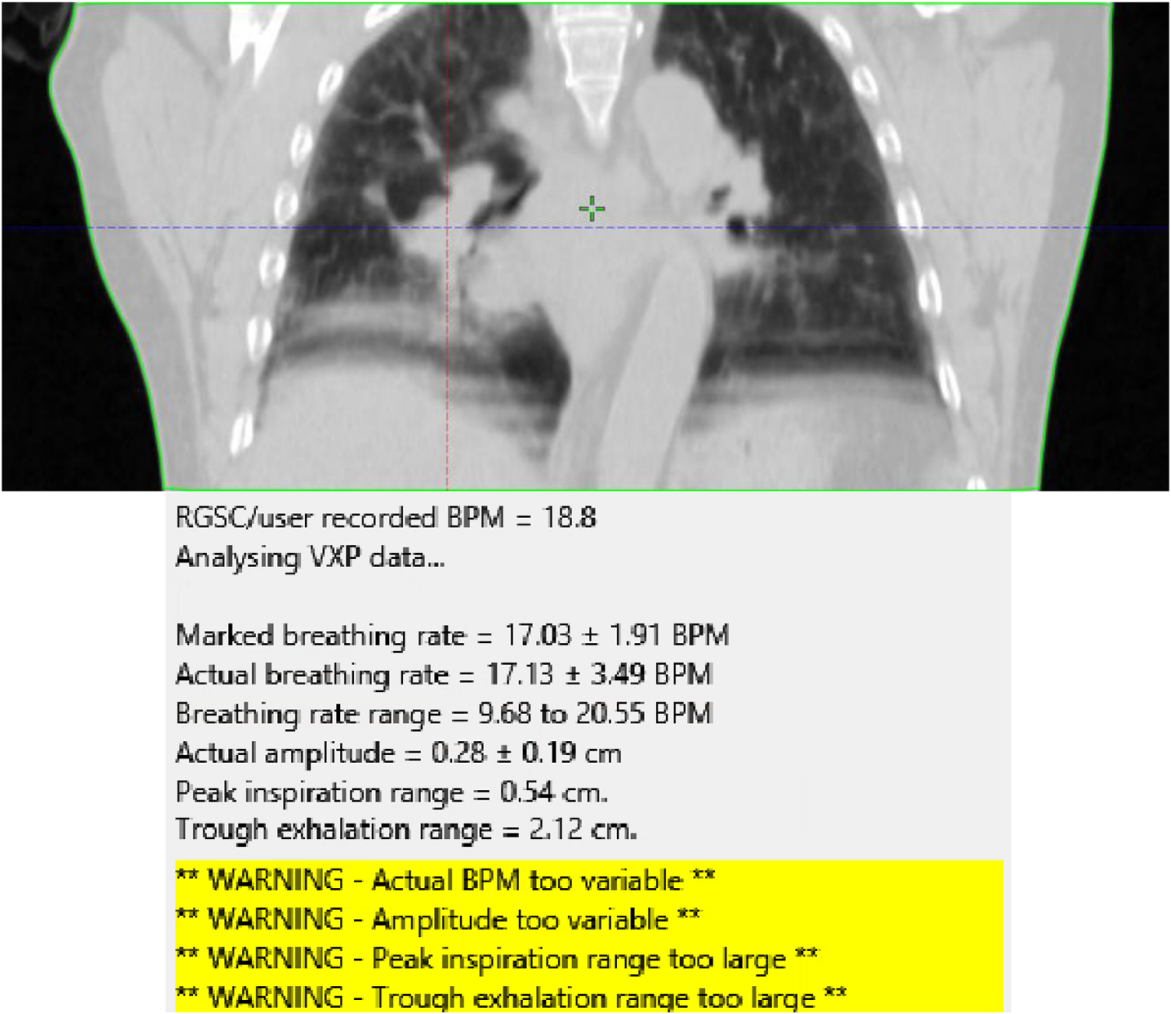
Case 2 example of poor 4DCT reconstruction with retrospective fail with FreeBreatheAudit. Breathing trace exceeded statistical tolerance (1 SD) for a number of metrics.

The second case that was identified as having a poor 4DCT reconstruction also did not have FreeBreatheAudit run while the patient was still on the CT couch and as such was only discovered once 4DCT images were imported into the TPS. FreeBreatheAudit was run retrospectively to see if this case would have been identified for re‐simulation. A coronal view of the dataset and the failed report are shown in Figure 10. This case also failed in multiple metrics resulting in misbinning of images and inconsistent HU seen on both single and average CT datasets. Error in breathing frequency was 20.35% (1 SD) and error in amplitude was 68%. The physicist in this case decided against using this dataset for planning and chose, in consultation with the RO, to use the 3DCT with appropriate margins. This case also highlights the need for immediate QA following acquisition of a 4DCT scan to catch artifacting as well as calculate uncertainties.

## 4 DISCUSSION

Analysis of breathing traces in the context of 4DCT simulation for a large patient study has not previously been published in the literature. Without analysis or quality assurance, breathing traces are used for 4DCT binning with no accurate quantification of uncertainties. The patient cohort in this study included raw breathing traces from 281 individuals, all taken from actual during‐CT‐scan simulations for treatment of lung targets. Previous studies concerned with 4DCT error have relied on data from only a small number of patients^12^, ^16^, ^17^ or manipulated patient breathing traces to suit the experiment.^7^ Due to the large patient dataset, average breaths per minute per patient display a Gaussian distribution with a mean of 15 BPM with 95% of cases between 6 and 24 BPM. Such a distribution from a patient set of this size has not been shown for 4DCT simulation. Since the patients were uncoached during simulation, this demonstrates the large variation in breathing habit and ability and thus the need for individual breathing trace quality assurance. The spread of breathing rates is also larger than the limits for 4DCT generation by some systems such as Siemens, furthering the need to flag issues with the breathing trace before treatment planning occurs.

Analysis of breathing trace files taken during patient simulation or treatment is not a new occurrence, with studies previously undertaken to compare treatment breathing to simulation in order to inform on coaching^17^ or to compare breathing monitoring systems.^10^ The latter study thus introduces the notion of breathing traces exhibiting error with RGSC demonstrating error improvements over RPM for irregular breathing.^10^ Our study has shown that even the value of BPM displayed on the RGSC screen to the operator has an uncertainty of around 20%. This may impact the accuracy of 4DCT dataset generation as the GUI value is input into many systems for use in its binning algorithm, so further supports the need for individual QA. In the case of irregular breathing, if one is to rewrite the VXP file with user determined max and min values instead of those set by RPM using its predictive method, generated 4DCT datasets can be seen to be much more accurate with smooth internal structures.^18^ Breathing irregularity is certainly more difficult for RGSC to trace properly with phantom studies demonstrating measurable resulting distortions in target shape, position, and density.^9^ However, these studies are either phantom based or only include data from a small number of patients. There has not been a study yet that takes a large sample of patient data taken during simulation and analyses the accuracy of their individual breathing traces to inform on the overall expected uncertainties of multiple breathing parameters for any given subsequent patient.

The work in this study has shown that patient breathing is variable in both phase and amplitude, even after pre‐simulation coaching. On average, the breathing frequency (BPM) had a standard deviation of at least 30%. This has not been shown before for a large patient sample. VXP inaccuracy with marking of minima and maxima is in agreement with studies by Wang et al.^18^ and Shi et al.^10^ The uncertainty in frequency within the scan due to breathing rate variation is 37% with the average difference between marked and actual frequency having a standard deviation of almost 10%. This is somewhat surprising since the operator can review and edit the peaks and troughs before VXP export. This further highlights the need for such a tool as proposed as, in practice, this step is either missed or not performed satisfactorily. Immediate breathing trace QA provides an efficient tool to flag poor breathing traces and consider re‐simulation while the patient is still on the CT couch. In addition to persons not correctly reviewing the breathing trace, actual (calculated in this study) frequency variations were less than that was marked which agrees with the conclusion by Wang et al. who found the predictive filter of RPM to incorrectly assign phase.^18^ Wang's study only featured a very small patient sample so this study improves upon those by testing the accuracy of the minima/maxima assignment more thoroughly through a larger patient sample, thus supplying better inference based on the breathing practices. This study is well positioned to supply uncertainties in breathing trace parameters for any subsequent patient.

Variations in assignment of phase (frequency errors) have direct clinical impact for patients receiving phase‐based treatment and should be quantified before treatment. Variations in amplitude were also assessed, however RGSC does not ‘mark’ amplitude in the same way as phase when phase binning is utilized so amplitude data was taken as accurate from the system. Of course, the amplitude values of the marker block are not the exact values for 1D movement of the target, though they have certainly been shown to be correlated, albeit with shifts in phase and amplitude and dependence on the marker placement.^19^, ^20^, ^21^, ^22^ ^]^ An assessment of amplitude variation from a large patient cohort has not been shown before with one of the most surprising results shown that patient inspiration and exhalation levels varied as much as the average amplitude of the whole trace. Uncertainty in amplitude due to breathing variation is 34%. Amplitude variations have direct clinical impact on tumor voluming and should be quantified for each new patient.

The clinical impact of breathing frequency and amplitude variability is well established in the literature^7^, ^8^, ^9^, ^11^, ^12^, ^23^, ^24^, ^25^ with various methods suggested to compensate for it during planning^26^ so it was not seen as a need to reinvent or re‐demonstrate this. However, what has not been shown is to apply these methods and calculations in the literature using the uncertainties calculated from breathing data of a large patient sample to inform on the likely dose errors in treatment. Dose error was calculated in this study for the hypothetical, worst case scenarios of (1) phase variability in our own calculation and (2) amplitude variation based on the methods of Cai et al.^12^ For our clinical cohort, almost all failures picked up by FreeBreatheAudit in this study did not progress to 4DCT reconstruction. At least qualitatively, the few failure case study patients that did proceed to 4DCT reconstruction and planning have aptly demonstrated the 4DCT dataset artifacts and errors that would be prevented through use of an individualized QA tool that assesses frequency and amplitude variability. This shows that performing QA can be used to pick up more qualitative errors and artifacts in 4DCT reconstruction as well as calculate uncertainties in scans that would otherwise appear well reconstructed.

For phase‐based/gated treatment, the recommendation according AAPM report TG142 is that the dose from gated treatment differ by no more than 2% compared to static.^27^ Phase and frequency uncertainty means that the target may be binned on the wrong phase with the difference in tumor position at the time of beam on depending on the amplitude and the phase (Table 3). For the realistic example case of a 2 cm volume, at worst, 66% of the PTV is missed with 100% of the GTV/CTV potentially missed if a margin were not utilized. The target is underdosed consistently due to the persistent phase‐based error, whilst surrounding normal tissues are overdosed. Though just a specific case, this is a greater dose difference compared with calculations made by Savanovic et al.^11^ for gated delivery with irregular breathing in a phantom. However, Sarker et al. observed a 50% error in motion for an irregular waveform which matches the phase offset calculated for our clinical scenario.^7^ Calculation of scenarios such as ours are only possible based on the knowledge of patient breathing variability as taken from a large patient sample. Since it is infeasible and irresponsible to add additional large margins in the direction of motion to cover binning uncertainty, phase errors in the breathing trace file should be quantified before planning in order to provide accurate calculations of dose and inform on possible re‐simulation of the patient.

With regard to the effect on tumor volume from amplitude variability, we know that the extent of tumor motion for irregular breathing traces tends to be underestimated^15^, ^24^ with smaller variations in amplitude corresponding to improved reconstructed images.^25^ Voluming error depends on amplitude variability and the length of the maximum intensity projection. From the set of 281 breathing traces, a voluming error of −11.41% was determined which is in good agreement with a smaller patient study performed by Sarker et al.^7^ Voluming error can now easily be estimated for any new patient based upon the uncertainties determined in this study.

Notwithstanding any additional phase‐based errors due to systematic time delays^16^, ^28^, ^29^ or partial projection artifacts due to intraphase residual motion,^6^ amplitude and phase variability has the potential to produce large dosimetric differences that will affect a patient's clinical outcome. Any new patient's breathing phase and amplitude uncertainty has been determined now based upon our large patient sample. Without individualized quality assurance, these uncertainties should be assumed and applied. Considering the magnitude of phase and amplitude uncertainty it is recommended that individualized quality assurance do be performed on a patient's breathing trace and 4DCT dataset in order to quantify potential dose errors, in the same way that each treatment plan undergoes quality assurance by the physicist. Recommendations are detailed in the following paragraphs.

For any new patient being simulated for treatment of the lung utilizing RGSC for 4DCT generation, we have well defined uncertainties (95% confidence) in VXP breathing parameters. This is summarized in Table 5. So even before individualized quality assurance the physicist and clinician should be well informed of the breathing trace uncertainties.

**TABLE 5:** Summarized breathing uncertainties for any new patient based on the data taken from simulations of 281 lung patients.

Parameter	Uncertainty (95%)	Figure/Table
Marked BPM (average)	19%	Table 2
Marked BPM (standard deviation)	37%	Table 2
Amplitude	34%	Figure 5
Inspiration level	7 mm	Figure 6
Exhalation level	9 mm	Figure 6
Tumor position (gated)	0−3.2 cm	Figure 8
Target volume	11%	Figure 4

An application should be written, similar to our FreeBreatheAudit, that can analyze VXP files and produce individualized quantifications of the parameters in Table 5 and of phase and amplitude variability, instead of relying on the values calculated in this study based on the patient sample. Tolerances should be decided upon by the physicist for each parameter, these can be statistical (based on the data in this study). When the application flags that there is a difference between a breathing trace metric identified by the application and that by RGSC/RPM, it is highly recommend that the resultant reconstructed 4DCT be carefully reviewed. More specifically, the user should carefully review all slices within the 4DCT for any obvious artifacts or distortions. Anecdotally, at our centers, FreeBreatheAudit is run straight after CT acquisition while the patient is still on the CT couch. If the application flags any failures, the breathing trace is reviewed and the patient rescanned. Review may involve double checking the peaks and troughs marked by RGSC as this is commonly missed by the operator. It is common for FreeBreatheAudit to catch variable patient breathing and require a rescan. This process always produces a 4DCT of satisfactory quality when followed. We have several cases of poor quality 4DCT datasets (qualitative analysis) being generated when this procedure has not been followed and FreeBreatheAudit not run, which has resulted in having to get the patient back in for re‐simulation.

The physicist should then calculate the possible dosimetric error for phase‐based treatment (if relevant) based on the methods in this paper. Any dosimetric error should be discussed with the radiation oncologist. Voluming error should also be calculated as shown in this study. Any voluming error that exceeds the defined target margins should also be discussed with the radiation oncologist. These recommendations should be followed before treatment planning starts in case the patient needs to come back for another simulation.

Some additional steps that will aid in producing accurate 4DCT images are: instead of contouring the ITV on the MIP images, which tend to mask errors in the reconstruction process, it is recommended that in this case it should instead be created by contouring the CTV/GTV on each individual phase. Subsequently, the delineated target on each phase should be checked for (1) volume consistency across different phases, acknowledging that there can be some volume difference towards the end of the breathing phase but not in the middle and (2) motion continuity, as the tumor motion between adjacent phases should be continuous. If the above process identifies any issues, recommendations should then be proposed to the radiation oncologist to consider other motion management techniques that are less susceptible to errors in 4DCT reconstruction, such as breath‐hold or abdominal compression techniques.

Several limitations in this study are acknowledged. Estimations of voluming error from amplitude variation are based on equations produced by Cai et al.^12^ which correlate perfectly with their phantom studies but only can be considered a ‘good’ fit for their patient study (*R* ^2^ = 0.76). This correlation is carried through to the estimation of uncertainties in this study. In addition, breathing traces were captured from CT beam‐on until final beam‐off and recorded breathing at moments between axial or helical exposures where the CT beam does not capture any imaging data. So, some breathing was analyzed that was not used in 4DCT reconstruction, however this was very minimal compared to the portion of breathing trace where the CT is actually capturing image data.

## 5 CONCLUSION

Currently, individualized quality assurance of patient breathing trace data is not featured in any international report or recommendation. CT simulation breathing traces from a large sample of 281 lung patients were analyzed to quantify inaccuracies in the RGSC system as well as uncertainties and variability in phase and amplitude‐based parameters. Analysis demonstrated differences between RGSC and calculated values, with total uncertainties in amplitude and phase making it possible to infer considerable differences in tumor volume and position. Without individualized QA or the assumption of large uncertainties determined in this work, significant errors go undetected that result in large dose differences for patients.

## AUTHOR CONTIBUTIONS

JR and YH conceived and designed the project. JR acquired the data. JR, YH, and KH analyzed and interpreted the data. JR, YH, and KH wrote and reviewed the paper.

## CONFLICTS OF INTEREST

None.

## Data Availability

The data that support the findings of this study are available on request from the corresponding author. The data are not publicly available due to privacy or ethical restrictions.
